# Motion Planning for Autonomous Vehicle Based on Radial Basis Function Neural Network in Unstructured Environment

**DOI:** 10.3390/s140917548

**Published:** 2014-09-18

**Authors:** Jiajia Chen, Pan Zhao, Huawei Liang, Tao Mei

**Affiliations:** 1 School of Engineering Science, University of Science and Technology of China, Hefei 230026, China; 2 Institute of Advanced Manufacturing Technology, Hefei Institutes of Physical Science, Chinese Academy of Sciences, Changzhou 213164, China; E-Mails: qiushui@mail.ustc.edu.cn (P.Z.); hwliang@iim.ac.cn (H.L.); tmei@iim.ac.cn (T.M.)

**Keywords:** autonomous vehicle, motion planning, radial basis function network, gradient descent method

## Abstract

The autonomous vehicle is an automated system equipped with features like environment perception, decision-making, motion planning, and control and execution technology. Navigating in an unstructured and complex environment is a huge challenge for autonomous vehicles, due to the irregular shape of road, the requirement of real-time planning, and the nonholonomic constraints of vehicle. This paper presents a motion planning method, based on the Radial Basis Function (RBF) neural network, to guide the autonomous vehicle in unstructured environments. The proposed algorithm extracts the drivable region from the perception grid map based on the global path, which is available in the road network. The sample points are randomly selected in the drivable region, and a gradient descent method is used to train the RBF network. The parameters of the motion-planning algorithm are verified through the simulation and experiment. It is observed that the proposed approach produces a flexible, smooth, and safe path that can fit any road shape. The method is implemented on autonomous vehicle and verified against many outdoor scenes; furthermore, a comparison of proposed method with the existing well-known Rapidly-exploring Random Tree (RRT) method is presented. The experimental results show that the proposed method is highly effective in planning the vehicle path and offers better motion quality.

## 1. Introduction

In the last few decades, both industry and academia have put enormous efforts in developing the technologies for autonomous road driving. The autonomous driving technology has ability to improve the safety, efficiency, energy consumption, and mobility in road driving [1]. Motion planning for autonomous vehicle is a core problem that is gaining importance in the research on autonomous driving in unstructured environments.

The objective of the motion planning is to compute a feasible and smooth path to reach a destination point without colliding with any obstacles [2,3]. The optimal path can meet many requests such as: collision free, shortest, maximize smoothness, or time-minimum.

There are various methods available for motion planning in the field of robotics in related literature. Heuristic planning methods [4–6], such as A*, are common solutions used to find the shortest path based on a certain decision criteria. However, a disadvantage of the A* algorithm is that the planning result in a grid-based configuration is rigid and it consists of a combination of straight lines.

In order to improve the efficiency of the classic methods, the probabilistic algorithm like Rapidly-exploring Random Trees (RRTs) has been developed. The RRTs employ the randomization process to efficiently explore the large state spaces and it can satisfy the vehicle's kinematic or dynamic requirements [7,8]. However, this method fails guarantee a safe distance between the vehicle and obstacles.

Many autonomous vehicles have demonstrated the motion planning ability to travel on the urban road at Defense Advanced Research Projects Agency (DARPA) urban challenge 2007. The vehicle “Junior” developed by Stanford applied a motion planning method based on a hybrid approach A*. This method assigns a continuous vehicle coordinate to each discrete cell, and the generated path can be realized by the actual robot [9]. Team AnnieWay developed a motion planning method that is based on a set of “tentacles” with different curvatures. The “tentacles” represent a set of pre-calculated trajectories defined in the ego-centered coordinate space of the vehicle. The route planner then selects the optimal “tentacle” as the generated path in real-time [10].

It is important to notice that motion planning algorithms of DARPA urban challenge teams are effective in the urban environment and these do not sufficiently prove to be useful in any unstructured environment. The main challenges in designing the motion planning algorithm for unstructured environment are: (1) the safety requirement, in the unstructured environment where the shape of the road is usually irregular, the generated path must guarantee a safe clearance from obstacles; (2) a limited sensing capabilities, such as range and perception accuracy, the planning method must be adaptive to the time-varying environment; (3) the nonholonomic constraint of vehicle.

With a framed-quadtrees data structure and an optimal algorithm, an online path planner was applied to incrementally re-plan optimal paths in outdoor mobile robots [11]. Based on Voronoi diagram, Garrido and Moreno invested a clearance-based shortest path planner [12]. In this paper, shortest path was firstly calculated by Dijkstra's algorithm. However, these methods are not suitable for autonomous vehicle motion planning because of the limitations of planning time and the dependency to the perception ability.

The Support Vector Machine (SVM) is used as an effective method to solve the motion-planning problem in unstructured environment. Qingyang *et al.* [13] proposed a method for unmanned ground vehicles by combining a basic path subdivision method for the topological maps of local environments and a SVM. The candidate routes boundary points are defined as positive and negative samples and SVMs are employed to train the separating surface. The smooth paths connecting the start and the destination points can be generated using the extended SVM. Huy *et al.* [14] proposed a path planning method for autonomous vehicle in cluttered environment with narrow passages. The RBF kernel SVM is used to maximize the safety margin for driving. The Lagrange multipliers of the SVM dual model are used to find the most critical points in the map and generate optimized hyperplane for the vehicle path. In the SVM-based planning methods, the stability of the generated path is largely influenced by the acquisition of margin data points. However in the complex environment, it is hard to construct a stable margin of roads depending on the existing sensors.

This paper addresses the problem of autonomous vehicle motion planning in the unstructured environment using the Neural Networks. The Neural Networks for Robot Motion Planning (RMP) was first used in [15]. In another study [16], the biologically-inspired general neural network approach has been applied to RMP for real-time collision-free motion planning in a dynamic environment. Dean A. Pomrleau proposed a three-layer back-propagation network designed for the task of road following used in the ALVINN system (Autonomous Land Vehicle In a Neural Network). The images from a camera and a laser range finder are used as the input of the network, the direction which the vehicle should travel is generated as the output. The test results showed that the autonomous vehicle can follow real roads under certain field conditions, successfully, with this method [17,18]. Based on neural networks, Boumediene and Chourqaui invested a collision-free path planner for moving robot among obstacles in partially structured environment. The simulation examples show that this method is effective [19]. This general model has been applied to point mobile robots, manipulator robots, car-like robots, and multi-robot systems [20–26]. In summary, the traditional planning methods do not explicitly consider that the expected collision-free path is always high-order and nonlinear in unstructured environment, while meeting the requirements of smoothness and real-time performance. In addition, some approaches rely on precise information of the environment. In a real-world scenario, there always exists inaccuracy in the description of the environment. The safety of autonomous driving will deteriorate when the perception error arises. The main contribution of this paper is a real-time navigation approach, based on the RBF network, which produces smooth and safe path for autonomous vehicle over large distances in real unstructured environment. In this method, the drivability grid map and global path are used in the selection of training data points and the generated path can timely react to the real environment.

The RBF network used for motion planning in unstructured environment possesses the following salient features [27–29]: (1) It is a universal approximator and possess the best approximation property. It is capable of approximating any nonlinear functions with high precision. The generated path can fit any road shape; (2) Considering the smoothness of the RBF, the generated path can be well executed by any autonomous vehicles; (3) Its learning rate is fast because of locally tuned neurons; therefore, the planning module can meet the real-time requirement of autonomous driving; (4) This method is not sensitive to the environment. The path always keeps a safe distance to the obstacles as the experiments show.

The remainder of the paper is organized as follows: Section 2 provides a brief introduction to the system architecture, especially the decision framework of the autonomous vehicle named “Intelligent Pioneer”. Section 3 describes the method of constructing the drivability map. Section 4 describes the RBF network and the learning algorithm, and then the simulation results with different RBF network parameters and a comparison of proposed method with higher-degree polynomial method are presented. The description of experiments, results, and future work is provided in Section 5. Finally, the conclusions are given in Section 6.

## 2. System Architecture

**Figure 1.**
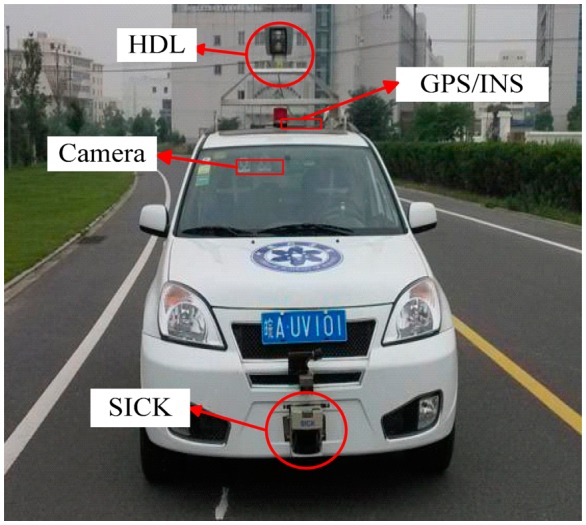
The autonomous vehicle named “Intelligent Pioneer”.

The autonomous vehicle named “Intelligent Pioneer” is built upon a 1.6 L Tiggo3 SUV made by Chery Automobile Co. The vehicle is equipped with two four-core computers and a suite of sensors including a GPS/INS receiver, three LIDAR sensors (two Sick LMS, one Velodyne HDL-64), and three cameras [30]. Figure 1 shows the sensor configuration of the intelligent vehicle.

The system architecture of “Intelligent Pioneer” is a distributed architecture [31]. It can be divided into five subsystems: environment perception system, decision making system, sensor system, control system, and the actuators. These subsystems are connected through Ethernet for inter-subsystem communication.

The perception system uses the three-dimensional laser radar (Velodyne) to model the complex environment of urban road and extract the road boundaries. The camera is used for lane detection, and two-dimensional laser radar (SICK) is used for the detection of static obstacles. As a whole, the perception system generates a data grid map with 512 × 512 grid cells, in which resolution is 0.2 *m* × 0.2 *m*.

RNDF (Road Network Definition File) contains geometric information on lanes, lane markings, stop signs, parking lots, and special checkpoints. MDF (Mission Definition File) consists of checkpoints and it decides the order of checkpoints.

**Figure 2.**
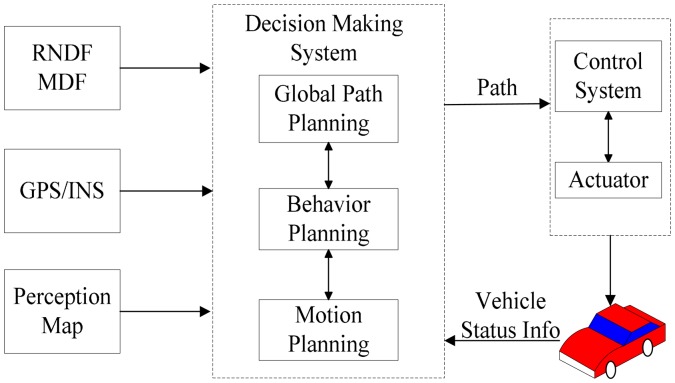
The software architecture of decision-making system.

Intelligent decision is a core problem in the study of autonomous vehicles. Based on the grid map provided by the perception system, the goal is to arrange the proper behavior and find a path without obstacles in complicated traffic environment, conforming to the rules of the road. A three-layer planning system consisting of global path, behavioral, and motion planning is used to drive in the urban environment, as shown in Figure 2. The output of decision-making system is a smooth path that consists of 200 two-dimensional (2D) points *P_i_* (*x_i_*, *y_i_*), *i* ∈ [0,199]; where *x_i_* is the latitude of the point, and *y_i_* is the longitude of the point. The controller receives the path as input, and then calculates the control command.

The control system [32] is constructed over the Controller Area Network CAN-2.0B bus topology. The computer of control system receives the status information of vehicle through CAN bus, and send the control command to each controlled member to achieve the goals of controlling the turn, brake, accelerator, gear, horn, and lights.

## 3. Construction of Drivability Grid Map

In order to apply RBF network, the effective data points are selected from the grid map provided by perception system. In this step, the digital map and generated global path is used as *a priori* knowledge.

**Figure 3.**
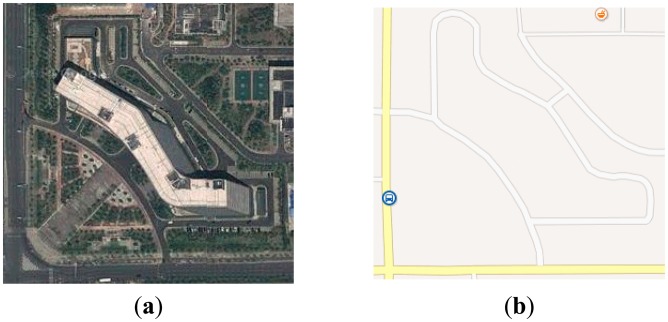

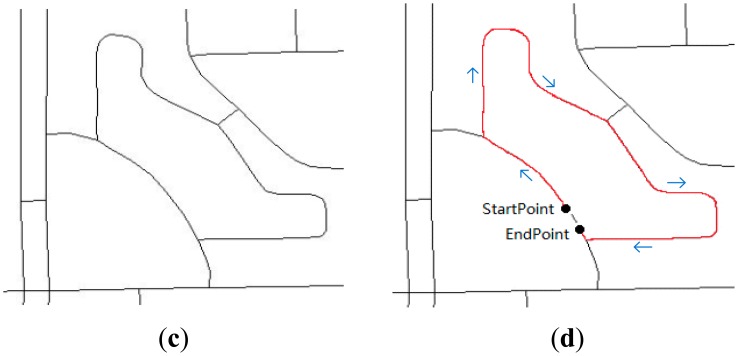
(**a**) Overhead view of the test area; (**b**) The digital map; (**c**) The road network constructed based on the digital map; and (**d**) The global path planning result.

The digital mapping is a process by which a collection of data is compiled and formatted into a virtual image. The primary function of this technology is to produce maps that accurately present particular areas detailing major road arteries and other points of interest. In this step, the points located on the roads and the intersections from the database of digital map are extracted to construct the road network map, as shown in Figure 3c. The A* algorithm is applied to obtain the global optimal path, as shown by the red line in Figure 3d.

**Figure 4.**
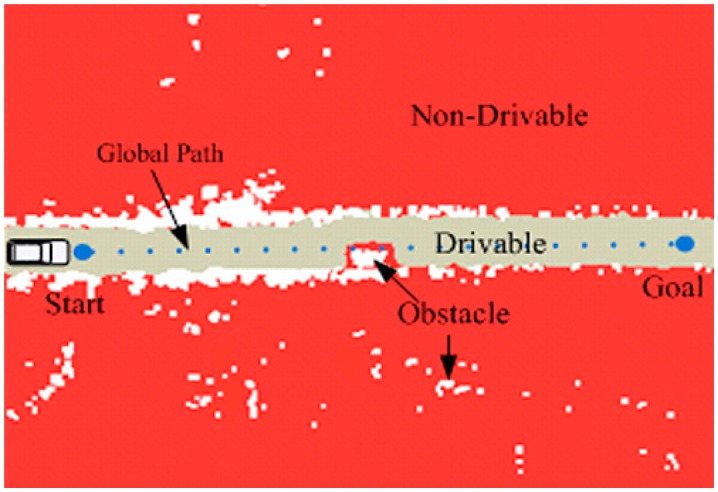
The drivability grid map.

In the motion planning application based on RBF network, the selection of training data points from the perception map is a necessary step; the drivability grid map is used to solve this problem, as shown in Figure 4. After generating the global path and mapping it to the grid map, the data points which are not occupied in the grid map from this path to the borders are selected. Based on these points, the drivable region is generated; this is shown as the gray region in Figure 4. All the perception data including the static and moving obstacles, drivable and non-drivable regions are rendered in the grid map. The locations of obstacles are detected by Velodyne. The blue points are global path interpolation result based on B-Spline. The red region represents the non-drivable region and the white points show the obstacles within the non-drivable region. The sampling points are taken as inputs from the drivable region for RBF network training. More importantly, this planning method does not require accurate information about the environment; furthermore, the planning result is not influenced by the minor change of drivable region.

## 4. RBF Network for Motion Planning

The RBF network behaves like a local approximation neural network and offers several advantages. The RBF network features a faster training compared to the back propagation network. It is less susceptible to the problems associated with non-stationary inputs due to the behavior of the radial basis function hidden units. In comparison to the sigmoid or S-shaped activation function used in back propagation, the hidden units in RBF network use a Gaussian or other similar basis kernel function. Each hidden unit acts as a locally tuned processor that computes a matching score between the input vector and its connection weights or centers. The weights connecting the basis units to the outputs are used to derive the linear combinations of the hidden units in order to produce the output.

### 4.1. The Structure of RBF Network

**Figure 5.**
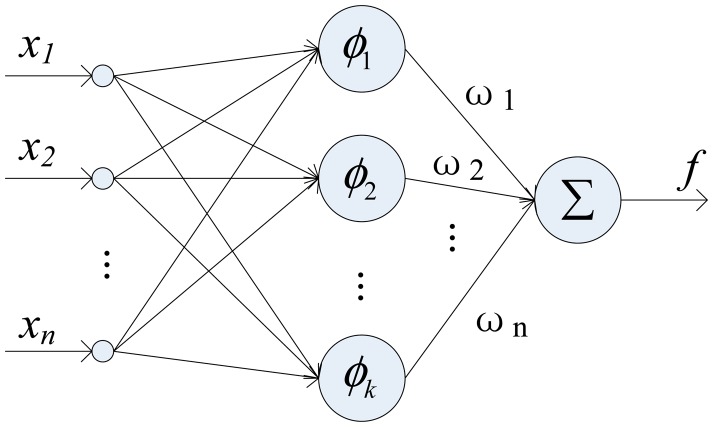
The structure of the RBF network.

The basic structure of RBF network consists of three distinct layers: an input layer, a hidden layer with a non-linear RBF activation function, and a linear output layer, as shown in Figure 5.

The inputs of hidden layer are the combinations of the input vector *x* = [*x_1_*, *x_2_*,…, *x_n_*]^T^. The incoming vectors are mapped over the radial basis functions in each hidden node. The output layer yields a vector *y* by linearly combining the outputs of the hidden nodes to produce the final output. The network output can be obtained by:

(1) $$y=f(x)=\sum_{i=1}^{k}\omega_{i}\phi_{i}(x)$$

where, *ω_i_* is the weight of *i*-th center, *φ_i_*(*x*) is some radial function, and *k* is the total number of hidden nodes. A radial basis function is a multidimensional function that describes the distance between a given input vector and a pre-defined center vector. There are different types of radial basis functions used in related literature. A normalized Gaussian function is usually used as the radial basis function, it is given as:

(2) $$\phi_{i}(x)=\exp(-\frac{{\left\|x-u_{i}\right\|}^{2}}{2\sigma_{i}^{2}})$$

where *μ_i_* and *σ_i_* denote the center and spread width of the *i*-th node respectively. There are many advantages of using the Gaussian function, some of the major advantages include:

- Simple representation for multiple input variables.
- Radial symmetry.
- Better smoothness.
- Highly analytical; thus, it is easy to carry on the theoretical analyses.

The output of network using Gaussian function is given as:

(3) $$y=f(x)=\sum_{i=1}^{k}\omega_{i}\exp(-||x-u_{i}|{|}^{2}/2\sigma_{i}^{2})$$

The Gaussian basis function is local to the center vector in the sense that:

(4) $$\underset{\left\|x\right\|\to \infty }{\lim}\rho \left\|x-u_{i}\right\|=0$$

This means that an RBF network with enough hidden neurons can approximate any continuous function with a wide range of precision value.

The application of all the existing data points as training set in the drivable region can lead to critical problems; some of the issues are listed in following:

- In general with a large training set, the solution of the matrix inversion function will be instable because of the large amount of conditions of hidden output matrix *H*, which is caused by the training data.
- The noises in samples will lead to over-learning, it is better to approximate the samples instead of interpolating.

**Figure 6.**
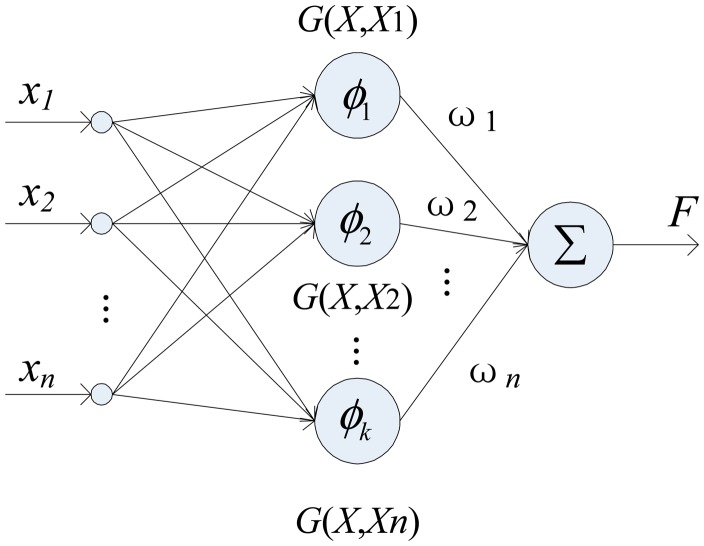
The structure of the regularization network.

To address those problems, the regularization network is used in this article. The structure of the network is shown in Figure 6.

The main advantages of regularization network are described in following.

- The regularization network is a universal approximator that can arbitrarily approximate any multivariate continuous function provided the availability of enough hidden units.
- The regularization network reaches the best approximation property. In this case, for each unknown nonlinear function *F*, there is always a choice of coefficients that approximates *F* better than all other possible choices.
- The solution computed by the regularization network is optimal. The optimality here means that the regularization network minimizes a functional that measures the deviation of the solution from its true value as represented by training data.

Assume the samples set is:

(5) $$s=\{(x_{i},y_{i})\in R_{n}\times R|i=1,2,\cdots N\}$$

The normal standard error term *E_s_*(*F*) is:

(6) $$E_{S}(F)=\frac{1}{2}{\sum_{i=1}^{N}\left(y_{i}-F(x_{i})\right)}^{2}$$

In this article, a term which constraint the complexity of approximation function is added based on the standard error term.

(7) $$E_{R}(F)=\frac{1}{2}{\left\|DF\right\|}^{2}$$

where *D* is the liner differential operator. The total error term of regularization network is defined as:

(8) $$E(F)=E_{S}(F)+\lambda E_{R}(F)$$

where the first term is used to control the precision of approximation function, the second term is called regularization term that controls the smooth degree of approximation function; λ is the parameter of regularization network. The solution of the above regularization problem can be derived as:

(9) $$F(x)=\sum_{i=1}^{N}\omega_{i}G(x,x_{i})$$

where *G*(*x*,*x_i_*) is the Gaussian function and ***ω****_i_* is the weight value. The larger curvature of the solution of regularization network *F*(*x*) results in a large value of ‖*DF*‖, consequently get damped with a higher factor.

(10) $$G(x,x_{i})=\exp(-\frac{1}{2\sigma^{2}}{\left\|x-x_{i}\right\|}^{2})$$

### 4.2. Learning Algorithm

Normally, the design and training of RBF network can be divided into the following three sections: computing the widths *σ_i_*, adjusting the centers *μ_i_* and adjusting the weights *ω_i_*.

In this paper the width is fixed according to the spread of the centers.

(11) $$\phi_{i}=e^{\left(-\frac{h}{d^{2}}{\left\|x-\mu_{i}\right\|}^{2}\right)},i=1,2,\cdots h$$

where *h* is the number of centers, *d* is the maximum distance between the chosen centers. Thus:

(12) $$\sigma =\frac{d}{\sqrt{2h}}$$

The smaller the value of *d* results in smaller width of RBF; it makes the base function more selective.

In this study, a single output RBF network-learning method with forgotten factor is used to calculate the centers and weights of the RBF network, as Algorithm 1 shows.



**Algorithm 1: RBFN( )**
1.Take a number of center *k*;2.**for**
*j* = 1,2,…,*k*
**do**3.Choose samples randomly as the center *μ_j_ = x_p_, p* ∼ *RAND(1, N)*;4.
$\sigma_{j}=\sigma =\frac{d}{\sqrt{2h}}$5.**end for**6.**for**
*i* = *1*,*2*,…, *n*
**do**7.**for**
*j* = *1,2*…, *k*
**do**8.Choose the weight value *ω_ij_* randomly, *ω_ij_* ∼ Rand(*ω_min_, ω_max_*)9.**end for**10.**end for**11.**while** error is more than 0.05 **do**12.add a sample (*x_p_, y_p_*)13.**for**
*i* = *1,2*,…, *n*
**do**14.calculate 
$\phi_{i}(x)=\exp(-\frac{{\left\|x-u_{i}\right\|}^{2}}{2\sigma_{i}^{2}})$15.**for**
*j* = *1,2*,…,*k*
**do**16.adjust weights17.
$\Delta \omega_{j}(t)=\eta \sum_{p=1}^{N}\beta_{p}e_{j}\phi_{j}(x_{p})$18.
$\omega_{j}(t+1)=\omega_{j}(t)+\Delta \omega_{j}(t)$19.**end for**20.**end for**21.**for**
*j* = *1,2*,…,*k*
**do**22.adjust center step23.
$\Delta \mu_{j}(t)=\eta \frac{\omega_{j}}{{r_{j}}^{2}}\sum_{p=1}^{N}\beta_{p}e_{j}\phi_{j}(x_{p})(x_{p}-\mu_{j})$24.
$\mu_{j}(t+1)=\mu_{j}(t)+\Delta \mu_{j}(t)$25.**end for**26.**end**
where *Φ_i_*(*X_j_*) is the output of the *i*-th hidden unit and η is the learning rate.

### 4.3. Simulations

In the simulation, the learning rate is set to 0.001 and the target error is set to 0.05. The numbers of hidden nodes is automatically determined by the numbers of sampling points. The algorithm is described in Algorithm 1.

**Figure 7.**
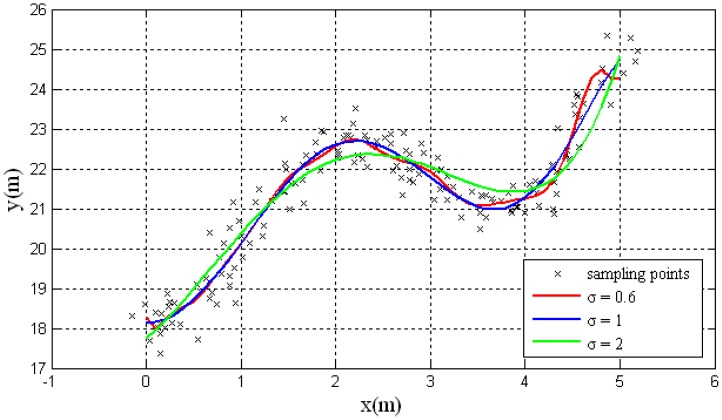
The fitting results of the RBF network with different parameters.

The simulation results are shown in Figure 7, the black points represents the road sampling data when simulating that the autonomous vehicle drives along a tortuous route. This sampling data is used in the training of RBF network training as the input.

According to Equation (13), the calculated expansion constant is 1, the other two network widths are chosen for comparison. The training results are shown with three different colors. The red solid line, blue solid line and green solid line are with three different values of σ, namely, σ = 0.6, σ = 1, and σ = 2 in each case.

From Figure 7, it can be observed that if σ is small, the training results have higher precision; however, the approximation will have a narrow peak at each data point resulting in a non-smooth observation. The large value of σ will ensure the smoothness of curve however at the cost of precision. The value of σ = 1 has proven an optimal performance with an average individual RBF.

**Figure 8.**
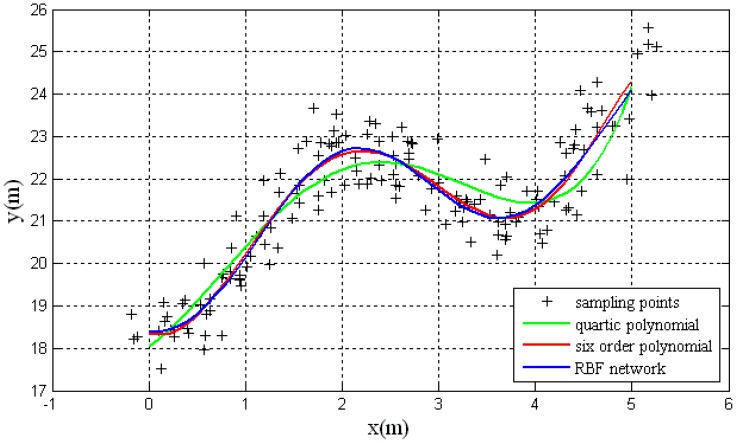
The fitting results of the RBF network and polynominal function.

For the same group of training samples, these samples can fitted with the method of higher-degree polynomial. The results are shown in Figure 8. The blue line shows the training result of RBF network, the green and red line represents the fitting results of quartic polynomial and six-order polynomial separately. In Figure 8, it can be seen that the fitting precision is higher when the order of polynomial is higher. The fitting result of six-order polynominal is similar to the training result of the RBF network.

**Figure 9.**
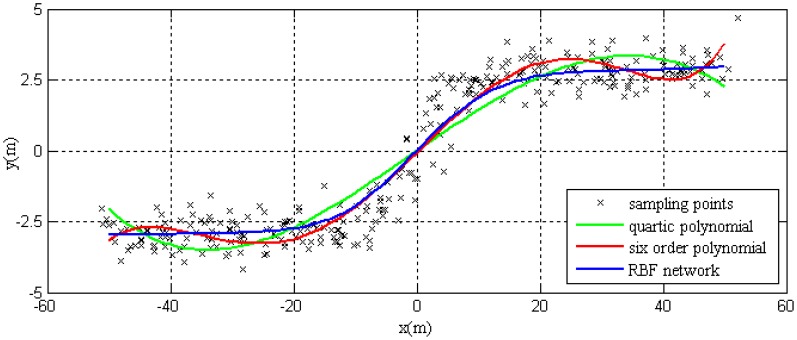
The fitting results of the RBF network and polynominal function.

However, the fitting with higher order polynomials is not applicable for autonomous vehicle driving in complex dynamic environment due to increased ill-conditioning. Figure 9 simulates the lane change behavior of autonomous vehicles.

As shown in this figure, the ill-conditioned polynomial makes the curve oscillatory. The higher order of polynomial results in a larger frequency of oscillation. While the training results of the RBF network still maintains a higher precision and smoothness. Hence, the motion planning method based on the RBF network is well adaptive to the complexity of driving environment.

## 5. Experimental Results and Discussion

In order to verify the effectiveness of the motion planning method, a real experiment is performed on “Intelligent Pioneer” and results are presented in this section. Two typical unstructured scenarios, such as zone and rural environments, are used in the experiments. In the experimental zone, a rotary course is designed. In the rural environment, the road has two lanes, each three meters wide. During these scenarios, the vehicle traveled at speeds up to 15 miles per hour, avoiding all the static and dynamic obstacles. Both the environments involved straight paths, curved paths, and obstacles. The sensed information around vehicle is changing all the time. Therefore, the results of the experiments can show the effectiveness of the real-time motion-planning algorithm.

**Figure 10.**
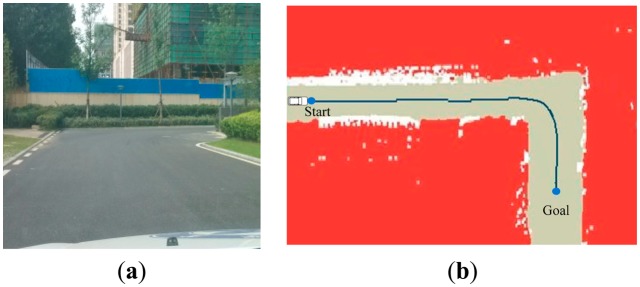
The experiment result in scenario 1. (**a**) shows a 90 degree turn in the zone; (**b**) shows the path generation result.

The planning experiments produced the grid maps plotted by program. The deep blue line in Figure 10 shows the planning result of a 90 degree turn in the zone. In this scenario, the waypoints of intersection are used to divide the sampling points into two groups and then put two paths together.

**Figure 11.**
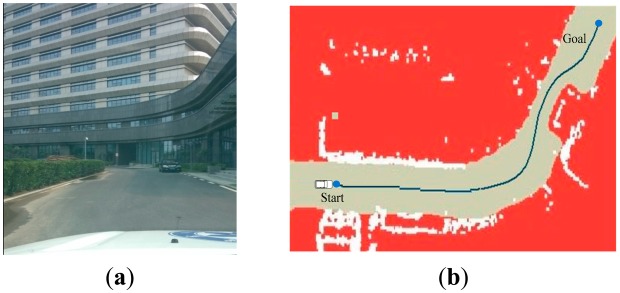
The experiment result in scenario 2. (**a**) shows the curve in the zone; (**b**) shows the path generation result.

**Figure 12.**
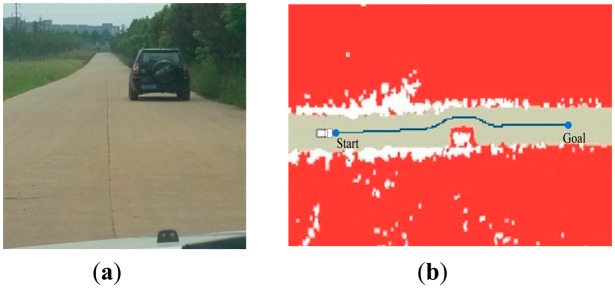
The experiment result in scenario 3. (**a**) shows navigating in the obstacle field; (**b**) shows the path generation result.

**Figure 13.**
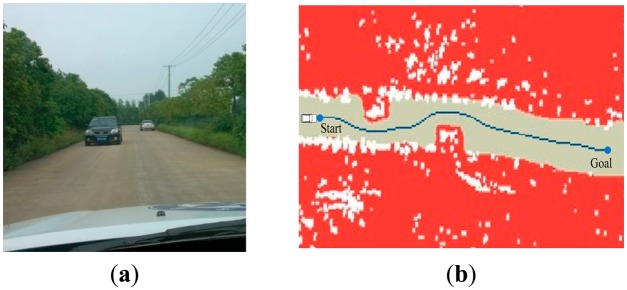
The experiment result in scenario 4. (**a**) shows navigating in the obstacle field; (**b**) shows the path generation result.

Figure 11 shows the result of curve in the zone. The path generated by RBF network is smooth and stable enough for “Intelligent Pioneer” to track. Figures 12 and 13 show navigating in the obstacle field. The deep blue line avoids the obstacles successfully and properly fits to the road shape.

**Figure 14.**
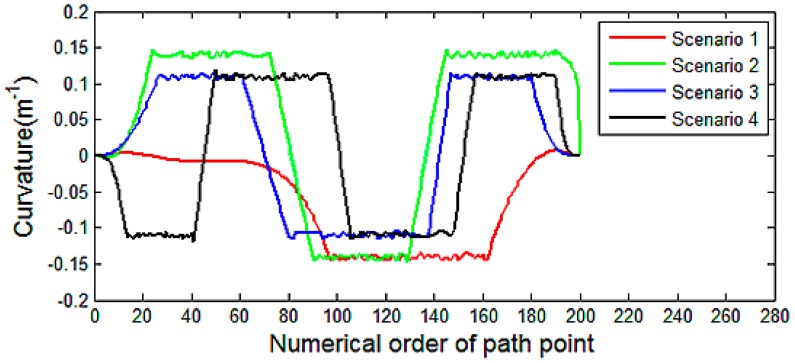
The curvatures of the generated path in different scenarios.

**Figure 15.**
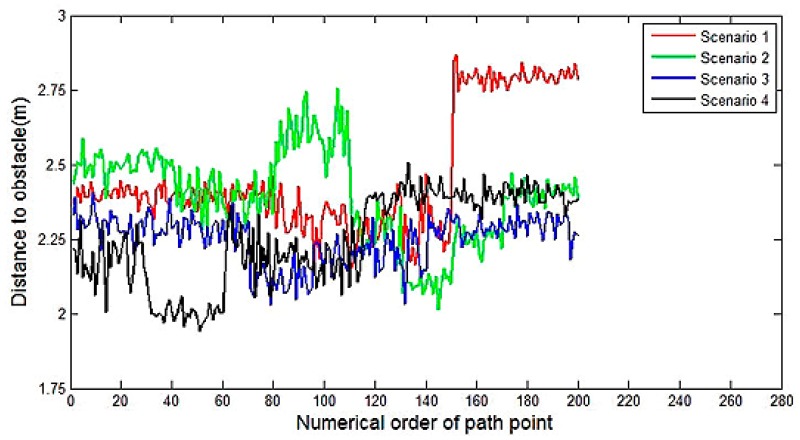
The distance to the nearest obstacle recorded in different scenarios.

The main evaluation indicators of motion planning results are listed in Table 1, which are generated based on Figures 14 and 15. It can be observed that, in these scenarios, the planning times are confirming to the requirement of real-time planning. The maximum turning curvature of Tiggo3 SUV is 0.19 *m*^−1^. The maximum curvatures of the generated paths meet the requirement of the vehicle's nonholomic constraint. As Figure 14 shows, the curvature is continuous so that the path is smooth. The small-scale jitter of curvature is caused by the discretization of perception grid map. Figure 15 shows that all the planning results have a safe clearance to obstacles, which enable the safe driving during experiment.

**Figure 16.**
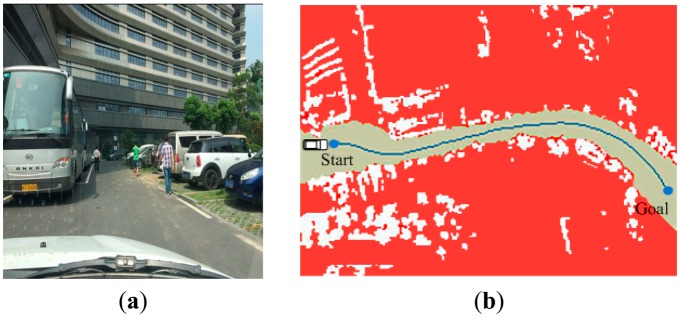
The experiment result in scenario 5. (**a**) shows navigating in the narrow space; (**b**) shows the path generation result.

To verify the motion planning system, the vehicle was put in the parking lot environment. The deep blue line in Figure 16b shows the planning result of the narrow space in the parking zone. This scenario containing large number of obstacles, such as vehicles and pedestrians, mean great increase in the complexity of the whole motion planning system. The deep blue line, which has a safe distance to the obstacles is smooth, safe, and easy to be followed.

**Figure 17.**
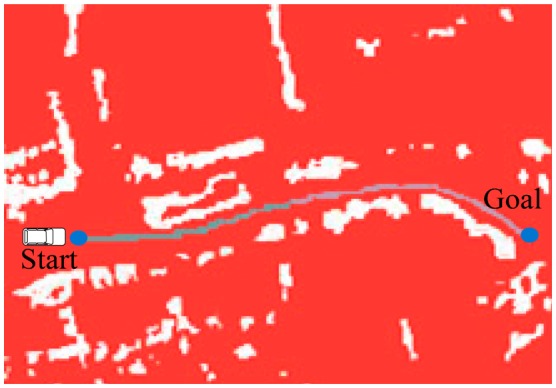
The path planning result of RRT+Bezier in scenario 5.

The proposed method is compared with the traditional RRT method in the scenario shown in Figure 16a. The path generated based on the RRT method is not smooth enough for vehicle to execute, therefore, the Bezier interpolation method is used to smooth it, as shown in Figure 17. As the RRT algorithm produces random results; therefore, the same experiment has been performed 500 times and the average characteristics are provided.

Table 2 shows the average planning time, minimum distance to the nearest obstacle for final path, and the maximum curvature. The planning time for the path based on the RBF network is less than the method of RRT+Bezier, they are both meeting the real-time planning requirements.

The maximum curvatures of both the methods satisfy the requirement of vehicle nonholonomic constraint. The path smoothness of RBF network and RRT + Bezier are almost same and both are able to generate a drivable path for vehicle to execute.

The minimum distance to the nearest obstacle for the path based on RBF network is smaller than the method of RRT + Bezier; therefore, it is much safer. This can be caused due to the fact that the Bezier interpolation method does not pass through the control point generated by RRT, therefore, the precision of path is influenced. In this narrow space, the distance between the path generated by RRT method and obstacles is small; the vehicle might be colliding with obstacles due to the control error.

**Figure 18.**
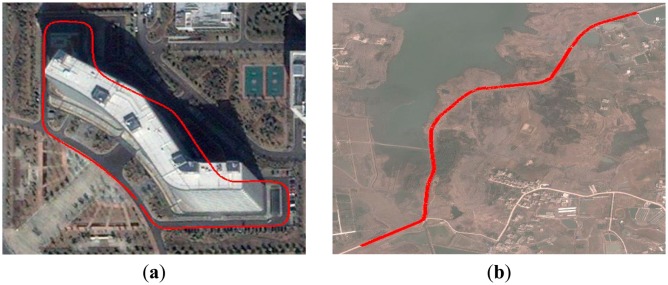
The GPS trajectory of “Intelligent Pioneer” recorded during experiment. (**a**) The red line indicates the trajectory recorded in the zone; (**b**) The red line indicates the trajectory recorded in the rural environment.

Figure 18 shows the GPS trajectory of “Intelligent Pioneer” encoded by SPAN-CPT. “Intelligent Pioneer” successfully achieved the goal of autonomously navigating through well-defined routes in zone and rural environment, validating the proposed approach.

## 6. Conclusions

This paper presented the RBF network algorithm, a neural network motion-planning algorithm specifically developed for autonomous vehicles and operating in uncertain, complex environments, such as rural environment or complex driving zones. By employing proper algorithm parameters, the planner can react intelligently and promptly to the new situations developed during the vehicle navigation. The path generated approximates any road shape with high precision, while satisfying the constraint of vehicle kinematic. The RBF is a kind of local approximation neural network with the advantage of fast learning speed; therefore, it can react fast to the environment and meet the real-time requirement of autonomous driving.

The experimental results indicate the suitability of this approach for autonomous vehicles navigation when a smooth and collision free path is possible. The autonomous vehicle reacts properly in the presence of obstacles, curves, and a 90 degree turn;

The complete motion-planning algorithm proved well-suited to tackle the challenges posed at the China Intelligent Vehicles Future Challenge (FC'2012). Its suitability for navigation in rural environment easily surpasses the demands of the Future Challenge.

Considering the online and sensor-based nature of the grid map environment model, it is believed that this motion-planning algorithm can also be applied to dynamic environments (with moving obstacles). The future work will focus on planning a path to allow dynamic obstacle avoidance, and the use of the algorithm in urban environment

## Figures and Tables

**Table 1.** The main evaluation indicators of motion planning results.

**IndicatorScenario**	**Planning Time (*ms*)**	**Minimum Distance to Obstacle (*m*)**	**Max Curvature(*m*^−1^)**
1	21.38	2.12	0.15
2	25.12	2.01	0.15
3	26.22	2.05	0.12
4	29.36	1.92	0.12

**Table 2.** The comparison of final path with two methods.

**ApproachIndicator**	**RRT + Bezier**	**RBFN**
Time (*ms*)	38.28	23.42
Max Curvature (*m*^−1^)	0.15	0.14
Minimum distance to obstacle (*m*)	0.5	0.9
